# Lyn modulates Claudin-2 expression and is a therapeutic target for breast cancer liver metastasis

**DOI:** 10.18632/oncotarget.3269

**Published:** 2015-03-25

**Authors:** Sébastien Tabariès, Matthew G. Annis, Brian E. Hsu, Christine E. Tam, Paul Savage, Morag Park, Peter M. Siegel

**Affiliations:** ^1^ Goodman Cancer Research Centre, McGill University, Montréal, Québec, Canada, H3A 1A3; ^2^ Department of Medicine, McGill University, Montréal, Québec, Canada, H3A 1A3; ^3^ Department of Biochemistry, McGill University, Montréal, Québec, Canada, H3A 1A3; ^4^ Department of Oncology, McGill University, Montréal, Québec, Canada, H3A 1A3

**Keywords:** breast cancer, liver metastasis, claudins, Src family kinase, Lyn

## Abstract

Claudin-2 enhances breast cancer liver metastasis and promotes the development of colorectal cancers. The objective of our current study is to define the regulatory mechanisms controlling Claudin-2 expression in breast cancer cells.

We evaluated the effect of several Src Family Kinase (SFK) inhibitors or knockdown of individual SFK members on Claudin-2 expression in breast cancer cells. We also assessed the potential effects of pan-SFK and SFK-selective inhibitors on the formation of breast cancer liver metastases. This study reveals that pan inhibition of SFK signaling pathways significantly elevated Claudin-2 expression levels in breast cancer cells. In addition, our data demonstrate that pan-SFK inhibitors can enhance breast cancer metastasis to the liver. Knockdown of individual SFK members reveals that loss of Yes or Fyn induces Claudin-2 expression; whereas, diminished Lyn levels impairs Claudin-2 expression in breast cancer cells. The Lyn-selective kinase inhibitor, Bafetinib (INNO-406), acts to reduce Claudin-2 expression and suppress breast cancer liver metastasis.

Our findings may have major clinical implications and advise against the treatment of breast cancer patients with broad-acting SFK inhibitors and support the use of Lyn-specific inhibitors.

## INTRODUCTION

The liver is a common metastatic site for numerous cancers, with the most prominent source of hepatic metastases originating from colorectal and breast tumors [1]. The formation of liver metastases has a major impact on cancer-related survival due to the vital functions carried out by this organ. Excluding brain metastases, the development of liver metastases is associated with the poorest outcomes relative to loco-regional, bone or lung metastases [2]. Cancer cells that arrive in the liver must contend with unique micro-environmental influences that differ markedly from the primary tumor. These include interactions with specialized cell types within the liver such as sinusoidal endothelial cells, Kupffer cells, Hepatic stellate cells, Pit cells and hepatocytes. Coupled with these new cellular interactions, cancer cells also encounter unique features of the liver architecture that together play significant roles in modulating the ability of cancer cells to seed, colonize and grow in this organ [3]. Together, these parameters greatly influence the selection of cancer cells that are best suited to thrive in the liver.

Recently, components of tight-junctional complexes have emerged as key modulators of the metastatic process [4, 5]. The main functional components of tight junctions are composed of claudin family members [4], which are also gaining increasing attention as metastatic regulators [6]. In colorectal cancer, Claudin-2 levels are elevated and its expression can be detected in pre-neoplastic conditions, such as inflammatory bowel disease that pre-dispose to colon cancer formation [7, 8]. More recently, Claudin-2 has been identified as an important positive modulator of colon cancer tumorigenicity [9, 10]. Furthermore, Claudin-2 expression has been reported in fibrolamellar hepatocellular, colorectal and pancreatic adenocarcinomas, as well as in liver metastases derived from these cancers [7, 11–13]. In contrast, Claudin-2 expression has been reported to decrease in breast cancers of increasing tumor grade and stage, and low Claudin-2 levels are associated with lymph node metastasis [14, 15]. However, despite reduced expression in primary breast cancers, we have recently demonstrated that Claudin-2 functions as a key mediator of breast cancer metastasis to the liver [16, 17].

Our previous studies revealed that Claudin-2 expression was selected for in aggressively liver-metastatic breast cancer cells; whereas, the expression of other cell-cell adhesion molecules was decreased [17]. We further demonstrated that Claudin-2 is functionally involved in liver metastasis and highly expressed in liver metastases from breast cancer patients [16, 17]. The mechanisms through which Claudin-2 enhances breast cancer metastasis to the liver involve enhanced seeding and early-stage survival [16]. Indeed, Claudin-2 enables integrin-dependent tumor cell adhesion to the extracellular matrix components and integrin-independent tumor cell adhesion to hepatocytes [16, 17]. Finally, Claudin-2 has recently been described as a prognostic biomarker able to predict the likelihood of breast cancer recurrence specifically to the liver [18].

Little is known about the mechanisms that control Claudin-2 expression in solid cancers. The objective of our current study is to define the regulatory mechanisms controlling Claudin-2 expression in breast cancer cells.

## RESULTS

### Pan inhibition of c-Src family kinase activity enhances Claudin-2 expression in breast cancer cells

We previously demonstrated that Claudin-2 expression in murine triple-negative breast cancer cells promotes the formation of liver metastases [16, 17]. However, little is known about the mechanisms that govern Claudin-2 expression in breast cancer cells. A previous study has demonstrated that EGFR-dependent activation of the MEK/ERK signaling pathway stimulates Claudin-2 expression in colorectal cancer cells [19].

**Figure 1:**
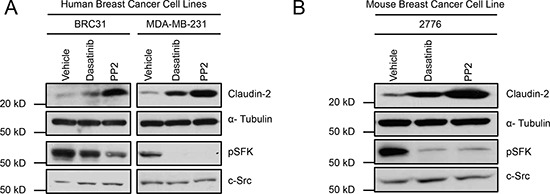
Inhibition of Src family kinases (SFK) enhances Claudin-2 expression in breast cancer cells Immunoblot analysis for Claudin-2 expression in both human breast (BRC31, MDA-MB-231) **(A)** or in a liver metastatic variant (2776) derived from the mouse 4T1 breast cancer cell line **(B)** treated with vehicle or two SFK inhibitors (Dasatinib and PP2). α-Tubulin served as a loading control and pSFK/Src blots revealed the efficacy of the SFK kinase inhibitors.

Signaling via the c-Src Family of non-receptor Kinases (SFK) has been reported to influence cancer cell morphology, adhesion, migration, invasiveness, proliferation, differentiation and survival. SFKs propagate numerous intracellular signals downstream of growth factor receptors, integrin complexes, steroid hormone receptors, G protein-coupled receptors and via interactions with components of the cytoskeleton [20, 21]. Indeed, activation of Src has been correlated with poor outcomes for patients with diverse types of cancer [22, 23]. Lyn, a SFK member, has recently received attention as an important regulator of signaling in basal/triple negative breast cancers. In this breast cancer subtype, Lyn expression correlated with poor survival and increased likelihood of recurrence [24, 25]. We have employed triple negative breast cancer models, expressing Claudin-2 (MDA-MB-231 and BRC31) [17, 26, 27], to elucidate the role of Claudin-2 as a promoter of the breast cancer liver metastatic phenotype [16, 17]. Thus, we investigated the involvement of SFKs in the regulation of Claudin-2 expression and liver metastatic ability of breast cancer cells. To accomplish this, we treated breast cancer cells with Dasatinib or PP2, two independent pharmacological pan-SFK inhibitors. We observed an increase in Claudin-2 levels when human (Figure 1A) and mouse (Figure 1B) breast cancer cells were individually treated with each SFK inhibitor.

We next assessed whether pan-SFK inhibitors affected transcription of *CLDN2* in breast cancer cells. In agreement with our immunoblotting results, quantitative real-time PCR showed that *CLDN2* mRNA levels are increased in both human and mouse breast cancer cells following treatment with pan-SFK inhibitors (1.73 – 3.33 fold induction for Dasatinib; 6.51 – 30.7 fold induction for PP2; Supplementary Figure 1A–1D). These results indicate that an SFK signaling pathway regulates *CLDN2* expression at the transcriptional level in breast cancer cells.

**Figure 2:**
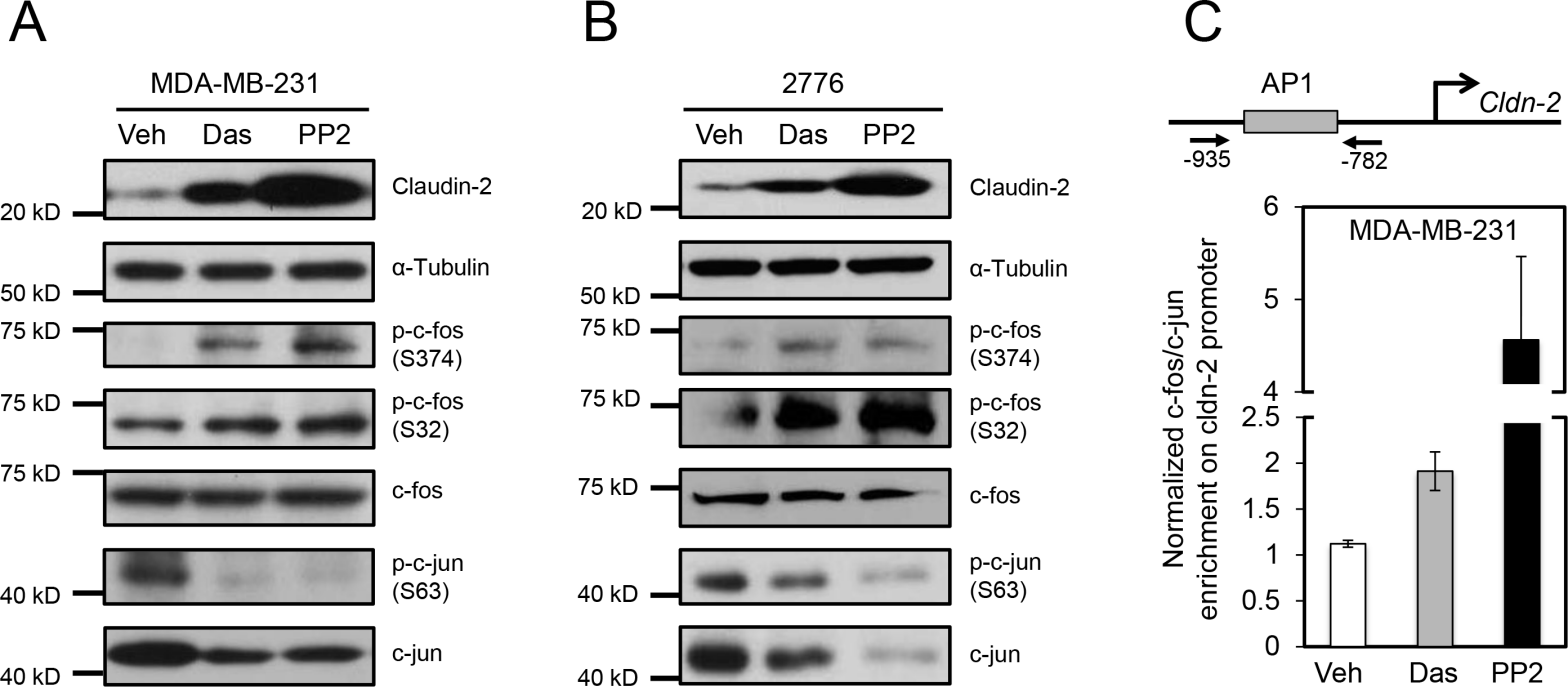
Differential phosphorylation and recruitment of c-Fos containing complexes to the AP1 site of the Claudin-2 promoter are associated with the changes in Claudin-2 expression following treatment with c-Src family kinase (SFK) inhibitors Treatment of human breast cancer cells (MDA-MB-231) **(A)** or the liver metastatic variant (2776) derived from the mouse 4T1 breast cancer cell line **(B)** with SFK inhibitors results in enhanced c-Fos phosphorylation (p-c-Fos) and elevated Claudin-2 expression. Diminished c-Jun phosphorylation (p-c-Jun) and total c-Jun levels are observed following treatment of breast cancer cells with SFK inhibitors. Immunoblots for α-Tubulin served as loading controls. **(C)** Chromatin immunoprecipitation experiments reveal that c-Fos/c-Jun complexes are enriched on the AP1 site within the *CLDN2* promoter in MDA-MB-231 breast cancer cells.

The EGFR-MEK-ERK1/2 pathway has been implicated in the transcriptional regulation of *CLDN2* in A549 lung adenocarcinoma cells through binding of the transcription factors, c-Fos and c-Jun, to the human *CLDN2* promoter region via an AP-1 binding site [28]. Phosphorylation of c-Fos (p-c-Fos) leads to stabilization of this transcription factor and enhanced transcriptional activity of the AP-1 complex [29]. Therefore, we assessed the effect of SFK inhibitors on the levels of p-c-Fos in breast cancer cells. Treatment of MDA-MB-231 breast cancer cells with Dasatinib or PP2 resulted in elevated levels of p-c-Fos (Ser374 and Ser32) and compared to total c-Fos levels, which remained unchanged (Figure 2A). Similar results were obtained using 4T1-derived mouse liver-metastatic breast cancer cells (Figure 2B). Interestingly, we observed a reduction in p-c-Jun (S63) and total c-Jun levels following treatment with pan-SFK inhibitors in both human (Figure 2A) and mouse (Figure 2B) breast cancer cells. These antibodies do not recognize JunB or JunD, raising the possibility that these Jun family members could heterodimerize with c-Fos.

We then used chromatin immunoprecipitation assays to monitor the recruitment of c-Fos to the human *CLDN2* promoter in MDA-MB-231 breast cancer cells following treatment with SFK inhibitors. As expected, a significant increase in c-Fos recruitment was observed at the *CLDN2* promoter in cells treated with inhibitors compared to controls (Figure 2C). These results demonstrate that SFKs act to suppress recruitment of c-Fos to the AP1 binding site within the human *CLDN2* promoter in breast cancer cells, which is relieved upon treatment with SFK inhibitors.

### Dasatinib treatment increases the formation of breast cancer liver metastases

**Figure 3:**
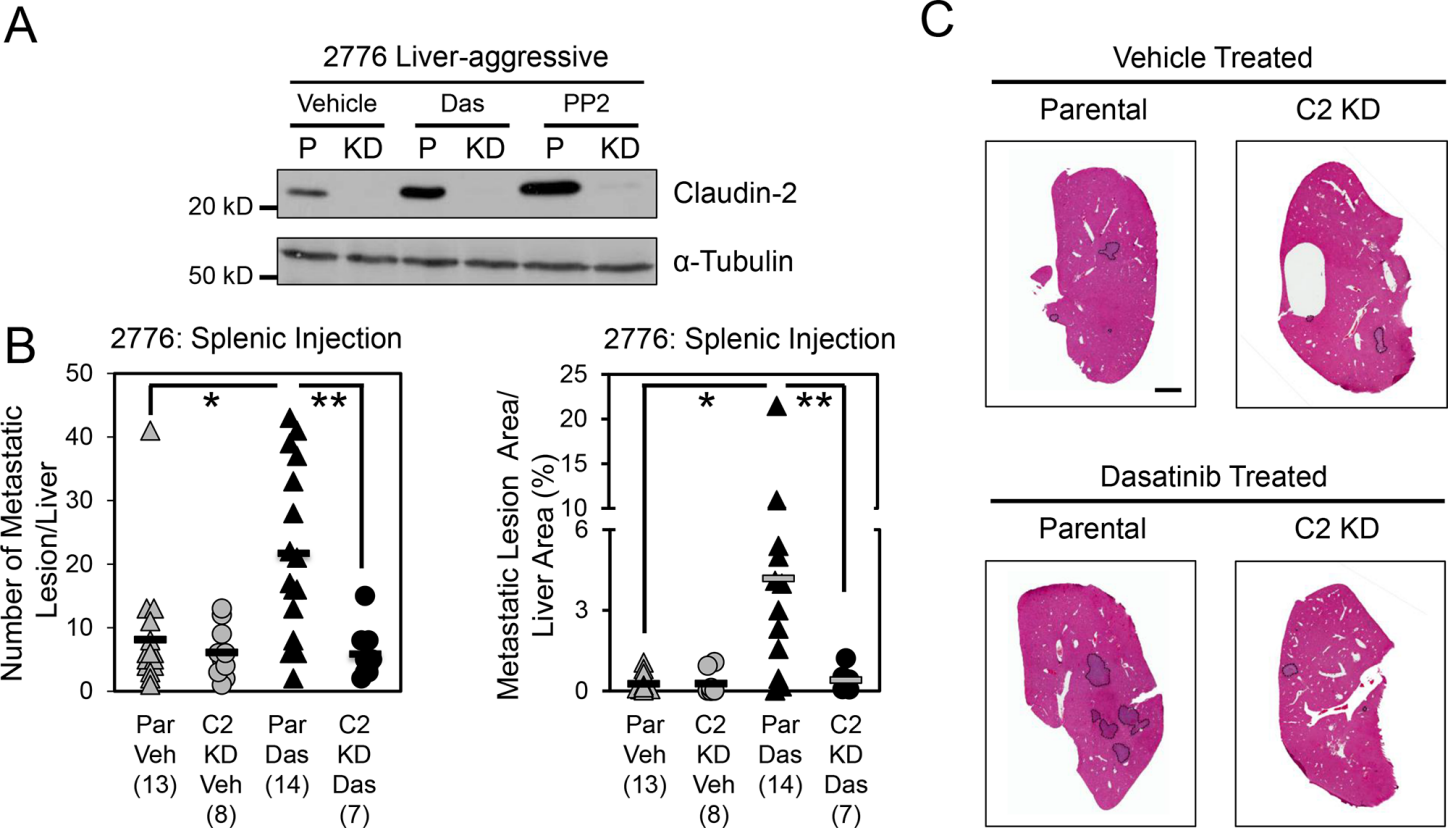
Dasatinib treatment enhances the formation of breast cancer liver metastases **(A)** Immunoblot analysis of Claudin-2 expression following treatment with c-Src family kinase inhibitors in parental 2776 cells (P) or 2776 cells harboring shRNAs against endogenous Claudin-2 (KD). As a loading control, total cell lysates were blotted for α-Tubulin. **(B)** Dasatinib treatment increases the number of metastases and the liver-metastatic burden derived from parental 2776 cells, but not from cells with diminished Claudin-2 levels, following splenic injection (1 × 10^4^ cells). A statistically significant increase in both the number of hepatic metastases or the liver metastatic burden is observed when the control cohort (Par Veh) is compared to the Dasatinib-treated cohort (Par Das) (*, *P* < 0.015). In contrast, this effect is lost when a cell population with diminished Claudin-2 expression was injected (C2 KD Das) (**, *P* < 0.012, Par Das vs C2 KD Das). The number of mice analyzed in each cohort is indicated in parentheses. **(C)** Representative images of H&E stained liver sections exhibiting the liver metastatic burden in each cohort. Scale bar represents 2 mm and applies to all panels. Par, Parental; Veh, Vehicle; Das, c-Src family kinase inhibitor (Dasatinib); PP2, Src family kinase inhibitor.

Given our previous data supporting Claudin-2 as an important promoter of breast cancer liver metastasis [16, 17], we assessed the effect of Dasatinib treatment on the formation of liver metastases following intra-splenic injection of 2776 liver-aggressive breast cancer cells that expressed endogenous Claudin-2 levels and 2776 cells that had stably reduced Claudin-2 expression (Figure 3A). We observed that mice treated with Dasatinib exhibited a 2.6-fold increase in the number of liver metastases and a 8.3-fold increase in the liver metastatic burden compared to animals receiving the vehicle control (Figure 3B and 3C). To determine if the increase in the liver metastatic burden was dependent on Claudin-2, we included a cohort of mice that received Dasatinib treatment but were injected with 2776 liver-aggressive cells harboring shRNAs targeting Claudin-2. Immunoblot analysis demonstrated that the presence of the shRNAs targeting Claudin-2 were able to almost completely suppress the increase in Claudin-2 expression that is normally seen following Dasatinib treatment (Figure 3A). Interestingly, mice injected with 2776 breast cancer cells harboring Claudin-2 targeting shRNAs failed to exhibit an elevated liver metastatic burden in response to Dasatinib treatment (Figure 3B and 3C).

To better understand the increase in the liver-metastatic burden following Dasatinib treatment, we examined the proliferative and apoptotic indices in the resulting lesions. We observed no differences in the degree of tumor cell proliferation (Ki67) in liver metastases derived from mice treated with vehicle or Dasatinib (Supplementary Figure 2A). Likewise, no statistically significant differences were observed in the number of apoptotic cells (Cleaved Caspase-3) within the liver metastases derived from these cohorts (Supplementary Figure 2B). However, in agreement with the increase of Claudin-2 expression following Dasatinib treatment that we observe *in vitro* (Figure 1B), immunohistochemical analysis revealed a clear increase in Claudin-2 positivity within hepatic metastases arising in the Dasatinib-treated cohort (Supplementary Figure 2C). Thus, the Dasatinib-induced increase in Claudin-2 expression may promote breast cancer survival and enhanced formation of liver metastases, as we have described previously [16].

### Diminished Lyn expression suppresses Claudin-2 levels while reduction of Fyn or Yes levels enhances Claudin-2 expression in breast cancer cells

**Figure 4:**
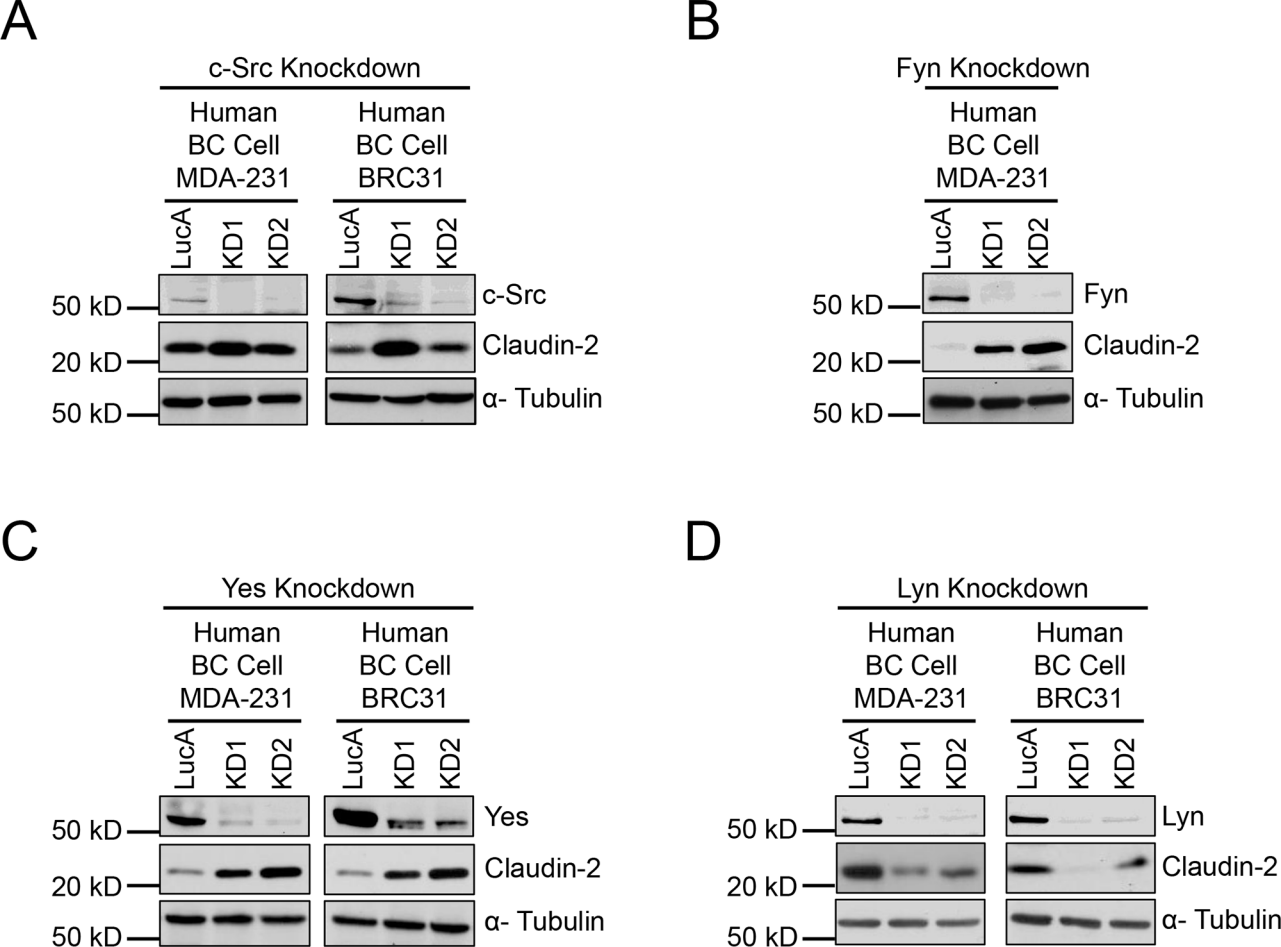
Reduction of Fyn or Yes expression increases Claudin-2 levels while reduced Lyn expression lowers Claudin-2 levels in breast cancer cells Immunoblot analysis of Claudin-2 expression in human breast cancer cells (MDA-MB-231 and BRC31) harbouring a control vector (LucA) or infected with two independent shRNA expression vectors (KD1 and KD2) against c-Src **(A)**, Fyn **(B)**, Yes **(C)** or Lyn **(D)**. As a loading control, total cell lysates were blotted for α-Tubulin.

Given the implication of these results for the treatment of breast cancer patients with pan-SFK inhibitors, we next investigated the involvement of individual SFK members (c-Src, Fyn, Yes or Lyn) in the regulation of Claudin-2 expression. To do so, we stably diminished the expression of each SFK in human breast cancer cells (MDA-MB-231 and BRC31) using shRNA-mediated approaches (Figure 4). No major effect on Claudin-2 levels was observed with single knockdown of c-Src (Figure 4A). However, individual loss of either Yes or Fyn, the latter not being expressed by BRC31 cells, resulted in a dramatic increase in Claudin-2 expression (Figure 4B and 4C), which recapitulates the effects seen with pan-SFK inhibitors (Figure 1A). Similar results were observed in the 2776 liver-aggressive cell population (Supplementary Figure 3A and 3B). Intriguingly, decreased Lyn levels significantly reduced Claudin-2 expression in human (Figure 4D) and mouse triple-negative breast cancer cells (Supplementary Figure 3C). We also observed that individual loss of Yes expression, and to a lesser extent Fyn, resulted in elevated Lyn levels in both MDA-MB-231 and BRC31 breast cancer cells (Supplementary Figure 4A and 4B). Taken together our observations reveal a critical role for Lyn in promoting Claudin-2 expression in breast cancer cells, highlighting the potential of this specific SFK family member as an important therapeutic target in the management of liver metastasis.

### Dasatinib (pan-SFK inhibitor) treatment enhances, while Bafetinib (Lyn-selective inhibitor) suppresses, Claudin-2 expression in breast cancer patient-derived xenograft explants

**Figure 5:**
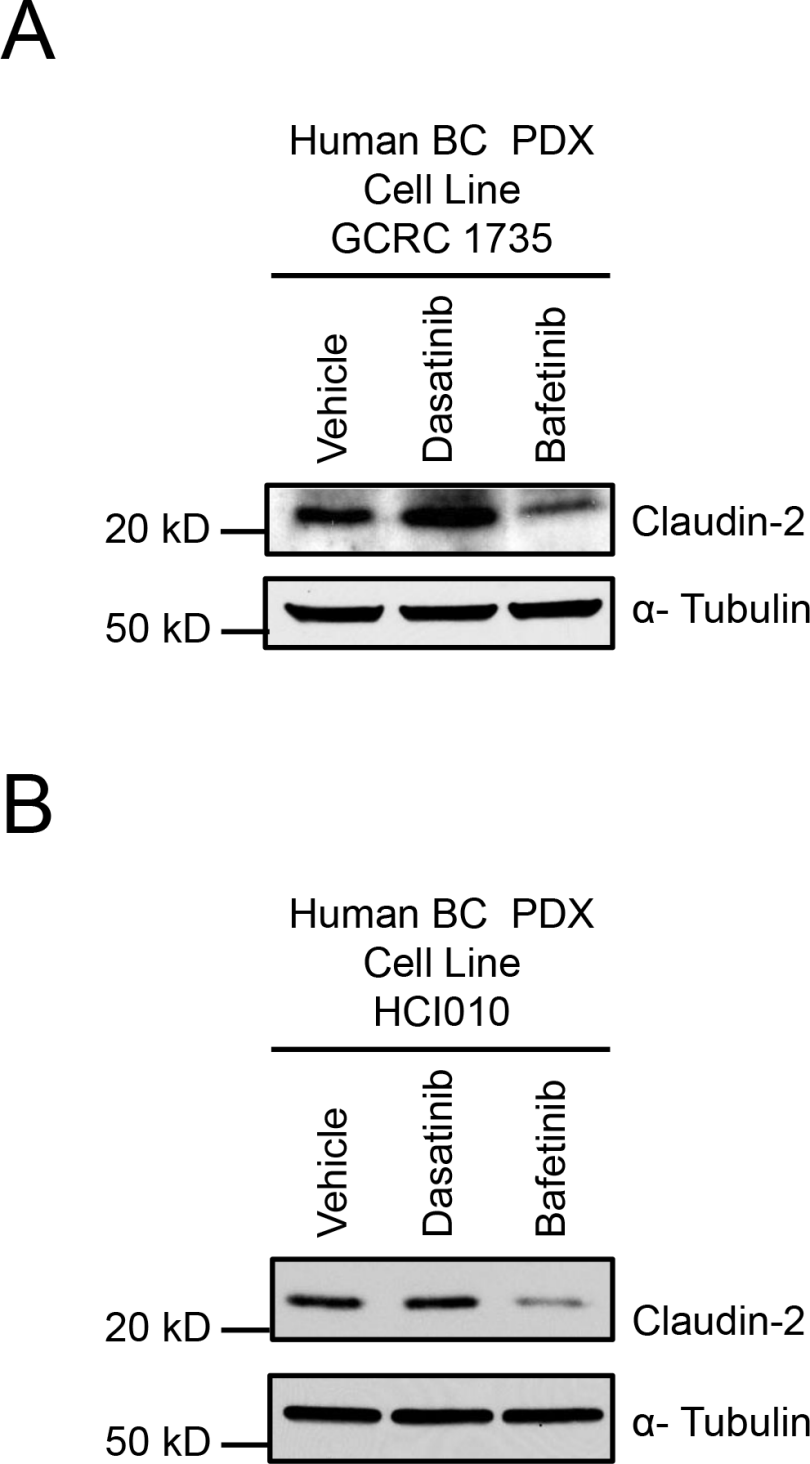
Dasatinib treatment increases, while Bafetinib treatment lowers, Claudin-2 levels in breast cancer patient derived xenograft explants Immunoblot analysis of Claudin-2 expression in GCRC1735 **(A)** or HCI010 **(B)** explants derived from triple negative breast cancer human patient derived following treatment with either Dasatinib (30 nM) or Bafetinib (10 μM) inhibitor. As a loading control, total cell lysates were blotted for α-Tubulin.

We next examined the potential relevance of our findings to additional human breast cancer models using a breast cancer patient-derived xenograft (PDX) developed in-house (GCRC1735) or a previously described triple negative breast cancer patient-derived xenograft (HCI010) [30]. As observed with human (MDA-MB-231 and BRC31) and mouse (2776) breast cancer cells (Figure 1), we observed an increase in Claudin-2 levels when GCRC1735 PDX cells were treated with Dasatinib (Figure 5A). However, no change in Claudin-2 levels was observed in the HCI010 PDX explant (Figure 5B).

Given the central role Lyn plays in regulating Claudin-2 expression, kinase inhibitors that specifically target Lyn could potentially reduce Claudin-2 expression and breast cancer liver metastasis. Bafetinib is a kinase inhibitor that is highly selective for Lyn and BCR/ABL [31–33] and exhibits little activity against other members of the c-Src Family, with the exception of Lck [34]. Interestingly, Bafetinib treatment dramatically reduced Claudin-2 levels in both the GCRG1735 or HCI010 PDX cultures. These data are in agreement with our results using shRNA-mediated knockdown of Lyn, which resulted in diminished Claudin-2 expression in both human and mouse breast cancer cells (Figure 4D; Supplementary Figure 3C).

### Bafetinib decreases the formation of breast cancer liver metastases

**Figure 6:**
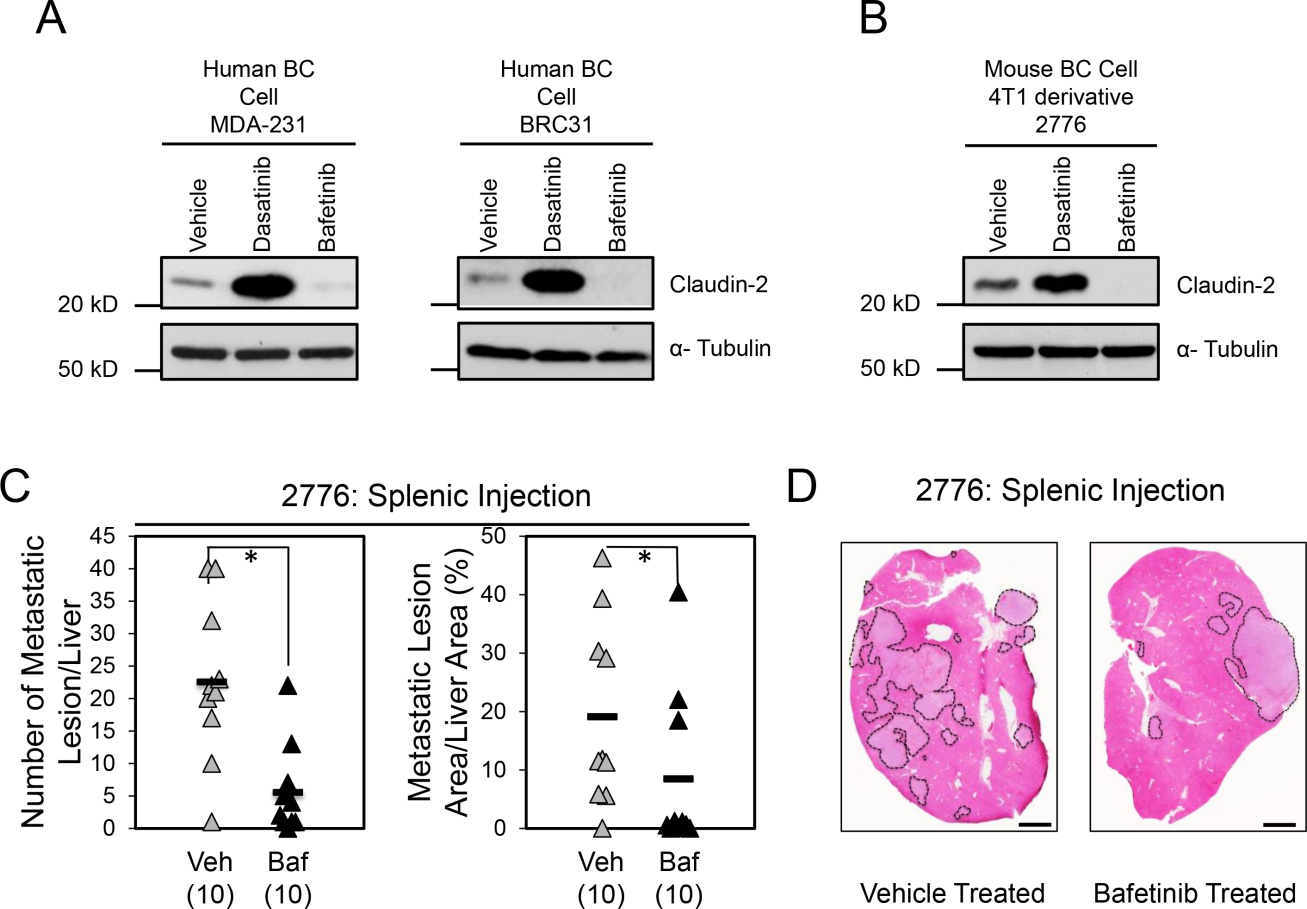
Bafetinib treatment impairs the formation of breast cancer liver metastases Immunoblot analysis of Claudin-2 expression in both human breast cancer cells (MDA-MB-231 and BRC31) **(A)** or 2776 mouse breast cancer cells **(B)** following treatment with Bafetinib inhibitor. As a loading control, total cell lysates were blotted for α-Tubulin (A, B). **(C)** Bafetinib treatment decreases the formation of liver metastases derived from 2776 cells following splenic injection (1 × 10^5^ cells). A statistically significant decrease in both the number of hepatic metastatic lesions or the liver metastatic burden is observed when the control cohort (Par Vehicle) is compared to the Bafetinib-treated cohort (Par Baf) (*, *P* < 0.05). The number of mice analyzed in each cohort is indicated in parentheses. **(D)** Representative images of H&E stained liver sections exhibiting the liver metastatic burden in each cohort. Scale bar represents 2mm. Veh, Vehicle; Baf, Lyn inhibitor (Bafetinib).

We also assessed whether Bafetinib treatment had the same effect on Claudin-2 levels in multiple breast cancer cell models. Uniformly, Bafetinib treatment reduced Claudin-2 levels in human breast cancer cells and liver-metastatic mouse breast cancer cells (Figure 6A and 6B). We next determined whether Bafetinib treatment would act to suppress the formation of breast cancer liver metastasis *in vivo*. Following intra-splenic injection of 2776 liver-aggressive breast cancer cells, we observed that mice treated with Bafetinib exhibited a 4-fold decrease in number of hepatic lesions and a 2.3-fold decrease in the formation of liver metastases compared to animals receiving the vehicle control (Figure 6C and 6D). We observed a slight decrease in tumor cell proliferation (Ki67) in hepatic metastases derived from 2776 liver-aggressive breast cancer cells that emerged in mice treatment with Bafetinib (Supplementary Figure 5A). In contrast, the degree of apoptosis (Cleaved Caspase-3) was unchanged in liver metastases that grew in the vehicle or Bafetinib-treated cohorts (Supplementary Figure 5B). Interestingly, the levels of Claudin-2 were clearly diminished in liver metastases from mice treated with Bafetinib compared to vehicle-treated controls (Supplementary Figure 5C), which recapitulate our *in vitro* findings (Figure 6A and 6B). Together, these observations suggest that, in contrast with the use of Dasatinib (Figure 3), breast cancer patients may benefit from treatment with Bafetinib to manage the formation of liver metastases.

Our previous experiments were conducted with one liver-aggressive cell population (2776) and the Dasatinib and Bafetinib experiments were conducted in separate cohorts. To confirm these observations, we assessed the effect of Dasatinib or Bafetinib treatment on an independent liver-aggressive breast cancer population (2792). As expected, we observed an increase in Claudin-2 levels when cells were treated with Dasatinib and a decrease in Claudin-2 levels in Bafetinib-treated cells (Supplementary Figure 6A). Following intra-splenic injection, mice treated with Dasatinib exhibited a 2.4-fold increase in the number of liver metastasis and a 3.3-fold increase in the liver metastatic burden compared to animals receiving the vehicle control (Supplementary Figure 6B–6D). In contrast, mice treated with Bafetinib exhibited a 2.1-fold decrease in the number of liver metastasis and a 3.6-fold decrease in the liver metastatic burden compared to the cohort that received vehicle alone (Supplementary Figure 6B–6D). Together, those observations suggest that treating breast cancer patients with Dasatinib (pan-SFK) could have the unanticipated consequence of promoting breast cancer liver metastasis formation whereas breast cancer patients may benefit from Bafetinib (Lyn-selective) treatment.

## DISCUSSION

Our previous studies demonstrated that Claudin-2 is functionally involved in breast cancer metastasis to the liver [16, 17] and a recent independent study has highlighted the potential of Claudin-2 as a prognostic biomarker that is able to predict the liver metastatic potential of primary breast tumors [18]. Our current findings reveal, for the first time, that signaling via Src family kinases can control Claudin-2 expression in breast cancer cells.

Using reporter assays and chromatin immunoprecipitation approaches, it has recently been shown that the EGFR-MEK-ERK1/2 pathway can regulate the transcription of *CLDN2* in A549 lung adenocarcinoma cells [28]. In these cells, activation of the EGFR pathway led to enhanced binding of the transcription factors, c-Fos and c-Jun, to the human *CLDN2* promoter region via an AP-1 binding site [28]. Intriguingly, increased AP-1 activity, induced by ectopic overexpression of c-Jun in SKBR3 breast cancer cells, resulted in a significant increase in liver metastasis following tail vein injection [35]. Our current results are in agreement with this mechanism as the application of pan-SFK pharmacological inhibitors increased the transcriptional activity of the AP-1 complex, as measured by the phosphorylation status of c-Fos (Figure 2A and 2B) and its recruitment to the AP-1 binding site of the human *CLDN2* promoter in breast cancer cells (Figure 2C). In contrast to lung adenocarcinoma cells or HER2+ breast cancer cells, we observed no evidence for a potential partnership role for c-Jun as the total levels of c-Jun and its phosphorylation (S63) were diminished in Dasatinib treated cells. Thus, it is conceivable that *CLDN2* expression in triple-negative breast cancer cells is controlled by an AP-1 complex composed of c-Fos and another Jun family member, such as Jun B or JunD. These data demonstrate that the transcription of Claudin-2 can be regulated through an SFK-c-Fos pathway.

Given the role of this SFK-c-Fos pathway in regulating Claudin-2 expression, we predicted that pan-SFK inhibitors would, in fact, enhance breast cancer metastasis to the liver, through the transcriptional up-regulation of Claudin-2. We demonstrate that Dasatinib administration promotes the liver metastatic ability of breast cancer cells in pre-clinical mouse models (Figure 3 and Supplementary Figure 6). The Dasatinib-induced increase in liver metastasis is mediated by Claudin-2 as knockdown of Claudin-2 completely abolishes the Dasatinib-mediated effect. We did not observe significant differences in cellular proliferation and apoptosis in liver metastases treated with Dasatinib, thus, it is likely that Dasatinib-induced Claudin-2 expression may enhance the early colonization steps of the liver-metastatic breast cancer cells as we have previously described [16].

These results are concerning given that SFK inhibitors have been employed as therapeutic agents in the setting of triple-negative metastatic breast cancer. A recent phase II study failed to demonstrate significant promising results that would justify the use of Saracatinib as a monotherapy in hormone receptor negative metastatic breast cancer [36]. In contrast, an independent phase II study has reported a modest clinical benefit with Dasatinib, which resulted in a partial response (2 patients) or stable disease (11 patients) in 43 evaluable patients with advanced triple-negative breast cancer [37]. Similarly, a phase II clinical study conducted in 70 patients with HER2-amplified tumors or estrogen receptor (ER) and/or progesterone receptor (PR)-positive tumors revealed that nine patients with ER+ and/or PR+ tumors had a partial response or stable disease when treated with Dasatinib [38]. Taken together, the results of several Phase II studies a revealed that Src inhibitors have shown little efficacy as a monotherapy, and combination strategies are now underway in the advanced/metastatic setting [39]. In these clinical trials, the endpoints used to assess the efficacy of Src inhibitors are focused solely on the primary tumor. Moreover, patients in these studies were typically followed for up to one year while receiving the inhibitor, with no long term follow up that would allow the potential impact of pan-SFK inhibitors on distant recurrences to be assessed, including the formation of liver metastases.

Pan-SFK inhibitors are known to target a broad spectrum of molecules. Dasatinib is an ATP competitive inhibitor of BCR-ABL, ephrin, c-KIT, PDGF receptor β, and targets all SFK members [40]. In our study, we have used concentrations below levels that result in off-target effects, suggesting that Dasatinib is likely targeting SFKs in our system [41]. PP2 is considered to be a more specific inhibitor, and has different off target effects, when compared to Dasatinib [42]. Together, these considerations argue that members of the SFK are the bone fide targets in breast cancer cells and raise the intriguing possibility that specific members of the SFK could be engaged differentially to regulate Claudin-2 expression, and the liver metastatic potential, in breast cancer cells.

Indeed, there are several SFK members, which include c-Src, Yes, Fyn, c-Fgr, Lyn, Lck, Hck, Blk, Yrk and Frk [43]. However, only a few of these SFK members are ubiquitous, including c-Src, Yes and Fyn; whereas others exhibit a more restricted pattern of expression in non-epithelial cells [43]. In breast cancer cells, loss of c-Src expression had no effect on Claudin-2 levels whereas diminished Yes or Fyn expression resulted in a dramatic upregulation of Claudin-2 levels. Thus, in the context of breast cancer cells, the dominant effect of PP2 or Dasatinib appears to be mediated through the inhibition of Yes or Fyn activity, which leads to higher Claudin-2 expression.

Through the use of Lyn-specific shRNAs and a Lyn-selective inhibitor, Bafetinib, we have identified Lyn as a potential therapeutic target to reduce liver metastatic ability of breast cancer cells. Our data comparing the effects of Dasatinib (a pan-SFK inhibitor) versus Bafetinib (a Lyn-selective inhibitor) suggest that specifically targeting Lyn would be recommended for the management of liver metastasis. Interestingly, Lyn has recently received attention as an important regulator of signaling in basal breast cancers, which represent the breast cancer models we have utilized in our current work, in which Lyn expression correlated with poor survival and increased likelihood of recurrence [24, 25]. Numerous clinical trials have been completed to assess the effectiveness of Bafetinib in treating glioma, hormone refractory prostate cancer or B-CLL patients (NCT01234740, NCT01215799, NCT01144260, NCT00352677). To date, none of these clinical studies have examined the potential effect of Bafetinib treatment on the development of distant liver metastasis nor are we aware of any ongoing trials that plan to examine the efficacy of Bafetinib in the context of metastatic breast cancer. Our results support the consideration of Lyn-selective inhibitors, such as Bafetinib, in the treatment of breast cancer liver metastasis.

## MATERIALS AND METHODS

### Cell culture

The 4T1 and MDA-MB-231 cell lines were obtained from the American Type Culture Collection (ATCC). The generation of 4T1-derived liver-aggressive cell populations has been described previously [17]. BRC31 human breast cancer cells were kindly provided by Dr. Rancourt and cultured as previously described [26].

As previously reported, all lentiviral shRNA vectors were retrieved from the arrayed Mission^®^ TRC genome-wide shRNA collections purchased from Sigma-Aldrich Corporation [44]. Additional information describing the shRNA vectors can be found at http://www.sigmaaldrich.com/life-science/functional-genomics-and-rnai/shrna/library-information.html or http://www.broad.mit.edu/genome_bio/trc/rnai.html, using the TRCN number. The following lentiviral shRNA vectors were used: shhuman*SRC*, TRCN0000038150 and TRCN0000199313; shhuman*LYN*, TRCN0000218210 and TRCN0000230901; shmouse*LYN*, TRCN0000023664 and TRCN0000023665; shhuman*FYN*, TRCN0000003097 and TRCN0000003101; shhuman*YES1*, TRCN0000010006 and TRCN0000121065. Lentiviral supernatants were generated as described at http://www.broadinstitute.org/rnai/public/resources/protocols. Pooled stable populations were maintained under 1.5 μg/ml puromycin antibiotic selection.

### Explant cultures

Explant cultures were derived from breast cancer patient-derived xenografts developed in-house (GCRC1735) or received as a kind gift from Alana Welm (HCI010) [30]. Briefly, excess breast tumor tissue from primary surgery was transported to the laboratory in ice-cold DMEM/F12, 50 μg/ml gentamicin, 1x penicillin-streptomycin, 2.5 μg/ml fungizone. Samples were cut into 1 mm^3^ fragments, covered in 50% matrigel, and transplanted into the mammary fat pad of 5–7 week-old NOG mice (Taconic, Hudson, NY, USA) under sterile conditions. When tumors reached 1 cm in the largest dimension, they were harvested aseptically, minced with sterile scalpels and dissociated at 37°C for 2–4 hours on a rotisserie in digestion medium (RPMI, 2.5% FBS, 10 mM HEPES, 1 mg/ml collagenase type IV, 50 μg/ml gentamicin), 3–5 minutes in 0.25% trypsin/EDTA and then passed through a 40 μm strainer. GCRC1735 breast tumor cells were cultured in adherent conditions, in which they were seeded on collagen I-coated plates in primary cell medium (DMEM, 5% FBS, 5 ng/ml human EGF, 5 μg/ml insulin, 1 μg/ml hydrocortisone, 35 μg/ml bovine pituitary extract, 50 μg/ml gentamicin). HCI010 breast tumor cells were cultured in non-adherent conditions, in which they were seeded on ultra-low attachment plates (Corning, Corning, NY, USA) in sphere medium (DMEM/F12, 1x B27, 20 ng/ml human EGF, 10 μg/ml insulin, 0.5 mg/ml hydrocortisone, 20 ng/ml bFGF, 10 μg/ml heparin, 50 μg/ml gentamicin). All human tissue was collected at McGill University Health Center in accordance with the protocols approved by the research ethics committee.

### Reagents

Dasatinib (LC Laboratories, Woburn, MA, USA), PP2 (calbiochem, Gibbstown, NJ, USA) and Bafetinib (Selleckchem, Houston, TX, USA) were prepared at the appropriate concentrations using DiMethylSulfOxyde (DMSO). For all *in vitro* assays, cells were treated for 18 hours using the following concentrations: Dasatinib: 30 nM; PP2: 10 μM; Bafetinib: 10 μM.

### Immunoblotting

Membranes were processed as previously described [17] and subjected to immunoblot analysis using the following antibodies: Claudin-2 (0.1 μg/ml; Cat. #: 325600; Invitrogen, Burlington, ON, Canada), phospho-Src family kinase (Tyr 416) (0.01 μg/ml; Cat. #: 2101), phospho-c-Fos (Ser 32) (0.035 μg/ml; Cat. #: 5348), Lyn (0.015 μg/ml; Cat. #: 2796), Fyn (0.07 μg/ml; Cat. #: 4023), Yes (0.02 μg/ml; Cat. #: 3201) (Cell Signaling, Whitby, ON, Canada), c-Src (0.4 μg/ml; Cat. #: 05–184; Millipore, Billerica, MA, USA), c-Fos (0.4 μg/ml; Cat. #: sc-253X), phospho-c-Fos (Ser 374) (0.2 μg/ml; Cat. #: sc-81485), c-Jun (0.4 μg/ml; Cat. #: sc-1694X), phospho-c-Jun (Ser63) (0.4 μg/ml; Cat. #: sc-822) (Santa Cruz Biotechnology, Dallas, TX, USA) and α-tubulin (0.5 μg/ml; Cat. #: T9026; Sigma, Oakville, ON, Canada). Blots were incubated with horseradish-peroxidase-conjugated anti-IgG secondary antibodies (Jackson ImmunoResearch Laboratories, Bar Harbor, ME, USA) and visualized with chemiluminescent HRP Substrate (Millipore, Billerica, MA, USA).

### Chromatin immunoprecipitation

ChIP assays were conducted as described previously [45]. A detailed protocol could be found in supplementary methods.

### *In vivo* analysis following Dasatinib or Bafetinib treatment

For experimental metastasis assays, parental 2776 liver-aggressive cells derived from the mouse 4T1 breast cancer cell line or 2776 cells harbouring shRNAs against endogenous Claudin-2 (1 × 10^4^ cells) were injected in the spleens of 6 to 8-week-old female Balb/c mice (Charles River, Senneville, QC, Canada) as previously described [17]. Dasatinib (10 mg/kg) was administered daily by oral gavage in 80 mmol/L citrate buffer, which was also used as vehicle control. Following splenic injection of the parental 2776 liver-aggressive cells (1 × 10^5^ cells), Bafetinib (10 mg/kg) was administered daily by oral gavage in 0.5% methylcellulose, which was also used as vehicle control. Mice were sacrificed 12 days later and the number of lesion as well as the metastatic area/tissue area was quantified using Imagescope software (Aperio, Vista, CA, USA) as previously reported [17].

The mice were housed in facilities managed by the McGill University Animal Resources Centre and all animal experiments were conducted under a McGill University approved Animal Use Protocol in accordance with guidelines established by the Canadian Council on Animal Care.

### Statistical analysis

Statistical significance values (*P* values) associated with liver metastasis formation from breast cancer cells injected in mice treated with Dasatinib (Figure 3) or Bafetinib (Figure 6) were calculated by performing a two-sample unequal variance student's *t*-test.

## SUPPLEMENTARY INFORMATION


