# Supplementary material for: Lyn modulates Claudin-2 expression and is a therapeutic target for breast cancer liver metastasis
Source: Oncotarget. 2015 Mar 25;6(11):9476–87. doi: 10.18632/oncotarget.3269 (PMC4496232; doi:10.18632/oncotarget.3269)
Supplement: Supplementary file 1 [file oncotarget-06-9476-s001.pdf]

SUPPLEMENTARY INFORMATION

SUPPLEMENTARY MATERIALS AND METHODS

Cell culture

The following lentiviral shRNA vectors were used to generate individual knockdowns in the 2776 liver-aggressive cell line: shmouse*FYN,* TRCN0000023380 and TRCN0000023381; shmouse*YES1*, TRCN0000023614 and TRCN0000023616. Pooled stable populations were maintained under 1.5 µg/ml puromycin antibiotic selection.

RNA isolation and real-time RT-PCR

Total RNA was extracted and reverse transcribed as previously described [1]. Following the reverse transcription reaction, all samples were diluted 1:50 in ddH_2O and subjected to real time PCR analysis with FastStart Universal Probe Master (Roche). Ten picograms of mouse *YES1* specific primers (*hCldn2* sense: 5′-GAGGTGGGAGTGGAGAATGG-3′; *hCldn2* antisense: 5′-GGCAAGAAGGTGGACATGTG-3′; *mCldn2* sense: 5′- TCCGTCTGTCTGTCTGTGTG-3′; *mCldn2* antisense: 5′- ATTGATCACCTTGTGTGTGC-3′; *YES1* sense: 5′-CACACCGGAAA ATCTTACAGAGC-3′; *YES1* antisense: 5′-TCCAAAAGGAGTCACCCCTGA-3′) were used in a total reaction volume of 15 µl. The following cycling conditions were used: 95°C for 10 minutes, followed by 40 cycles each consisting of 95°C for 15 seconds, 60°C for 30 seconds and 72°C for 45 seconds. Incorporation of SYBR Green dye into the PCR products was monitored using a Rotor Gene RG-3000 Real time PCR system (Roche, Laval, QC, Canada). Pfaffl analysis method was used to measure the relative quantity of gene expression [2]. The reference gene, *Gapdh* (*Gapdh sense*: 5′-CAAGTATGATGACATCAAGAAGGTGG-3′; *Gapdh antisense*: 5′-GGAAGAGTGGGAGTT GCTGTTG-3′) was selected based on its stable expression in all cell populations analyzed. Relative mRNA levels were expressed in terms of fold induction over the vehicle treated cell populations. All measurements were done in triplicate and three independent experiments were performed for each gene target.

Chromatin Immunoprecipitation

ChIP assays were conducted as described previously [3]. Briefly, $1x10^7$ MDA-MB-231 (vehicle-treated, treated with Dasatinib or treated with PP2) were cross-linked using 1% formaldehyde for 10 min at room temperature. Cells were then washed twice with ice-cold phosphate-buffered saline, collected, and centrifuged. Pellets were first washed once with buffer A (0.25% Triton X-100, 10 mM EDTA, 0.5 mM EGTA, 10 mM Tris-HCl, pH 8) supplemented with protease inhibitor mixture (1 mM sodium orthovanadate, 10 mM sodium fluoride, 10 µg/ml aprotinin, 10 µg/ml leupeptin, 1mM β-glycerophosphate and 2.5 mM sodium pyrophosphate). Cell pellets were then washed once in buffer B (200 mM NaCl, 1 mM EDTA, 0.5 mM EGTA, 10 mM Tris-HCl, pH 8) supplemented with protease inhibitor mixture. After incubation on ice for 30 minutes, cells were pelleted and resuspended in sonication buffer (0.5% SDS, 0.5% Triton X-100, 140 mM NaCl, 1 mM EDTA, 0.5 mM EGTA, 10 mM Tris-HCl, pH 8) supplemented with protease inhibitor mixture before being sonicated twice for 25 seconds using a VirSonic 100 (Virtis) sonicator. Soluble chromatin was diluted 2- fold in chromatin immunoprecipitation dilution buffer (1% Triton X-100, 2 mM EDTA, 150 mM NaCl, 10 mM Tris-HCl, pH 8) and immunoprecipitated for 16 hours using magnetic beads (Invitrogen, Burlington, ON, Canada) and c-Fos, c-Jun or isotype control antibodies (Santa Cruz Biotechnology, Dallas, TX, USA).

Following immunoprecipitation, beads were washed sequentially for 2 min each with buffer I (0.5% Nonidet P40, 1 mM EDTA, 10 mM Tris-HCl, pH 8, 150 mM KCl), with buffer II (0.5% Triton X-100, 10 mM Tris-HCl, pH 8, 150 mM NaCl) with buffer IIIA (0.5% Triton X-100, 10 mM Tris-HCl, pH 8, 400 mM NaCl), with buffer IIIB (0.5% Triton X-100, 10 mM Tris-HCl, pH 8, 500 mM NaCl) and with buffer IV (250 mM LiCl, 0.5% Nonidet P-40, 1 mM EDTA, 10 mM Tris-HCl, pH 8). Precipitates were then washed two times with TE buffer (10 mM Tris, pH 8.5, 1 mM EDTA) and eluted with 1% SDS, 10 mM EDTA, 10 mM Tris-HCl, pH 8. The eluates were incubated at 65°C for at least 16 h. RNAse A was then added and samples incubated for 1 hour at 37°C followed by a proteinase K treatment (50 µg, 55°C for 1 hour).

The isolated DNA fragments were purified using a PureLink quick PCR purification kit following the manufacturers instructions (Invitrogen, Burlington, ON, Canada). Real time quantitative PCR was performed using a LightCycler480 instrument with FastStart Universal Probe Master (Roche, Laval, QC, Canada) and specific primers for genomic regions as reported previously [4]. ChIP enrichment was normalized against the control region and further normalized against the IgG control. Each experiment was repeated three times. The primers used for ChIP-quantification were: −935S (sense: 5′-CCCTTAGT GTCCTGAATCTTGG-3′) and −782A (antisense: 5'-AAGAGTCTGCCACAGAGG AAAG-3′) for the AP1 binding site or − 1538S (sense: 5′-GCCAGATTGTG GTGGGTTGA-3′) and −1387A

(antisense: 5′-AGAGACCAGGTTTTGCCATGT-3′) for a non-AP1 binding site from the human *CLDN2* promoter.

Immunohistochemistry

Tissue fixation and immunohistochemical (IHC) staining was carried out as previously described [5]. The proliferative index in the liver metastatic lesions was assessed by staining with a Ki67 antibody (1 μg/ml; Cat. #: ab15580; Abcam, Toronto, ON, Canada). Anti-Cleaved-Caspase 3 staining (0.2 μg/ml dilution; Cat. #: 9661; Cell Signaling, Whitby, ON, Canada) was performed to quantify apoptosis within the hepatic lesions. Following incubation with the primary antibody, secondary biotin-conjugated antibody (Jackson Laboratories) was applied for 30 minutes. After washing with distilled water, slides were developed with diaminobenzidine (Dako) as the chromogen. All slides were counterstained using Harris haematoxylin before being scanned using a Scanscope XT digital slide scanner (Aperio). Quantification was performed by analyzing hepatic metastases with Imagescope software (Aperio) using IHC nuclear algorithms for Ki67 and Cleaved Caspase-3 staining. For quantification of Ki67 and Cleaved Caspase-3 staining, positively stained nuclei were represented as a percentage of total nuclei per field.

For Claudin-2 staining, tissue sections were stained using a Benchmark XT autostainer (Ventana Medical System Inc.) following manufacturer's instructions using pre-diluted antibody (1 μg/ml; Cat. #: ab76032; Abcam, Toronto, ON, Canada). Quantification was performed by analyzing hepatic metastases with Imagescope software (Aperio) using a modified protocol derived from the Aperio positive pixel count algorithm. Claudin-2 positivity was represented as a percentage of total pixels per region of interest.

In vivo analysis of liver metastasis formation following Dasatinib or Bafetinib treatment

For experimental metastasis assays, parental 2792 liver-aggressive cells derived from the mouse 4T1 breast cancer cell line (5×10^4 cells) were injected in the spleens of 6 to 8-week-old female Balb/c mice (Charles River, Senneville, QC, Canada) as previously described [1]. Dasatinib (10 mg/kg) was administered daily by oral gavage in 80 mmol/L citrate buffer, which was also used as vehicle control. Bafetinib (10 mg/kg) was administered daily by oral gavage in 0.5% methylcellulose, which was also used as vehicle control. Mice were sacrificed 14 days later and the number of lesions as well as the metastatic area/tissue area was quantified using Imagescope software (Aperio, Vista, CA, USA) as previously reported [1].

Statistical analysis

Statistical significance values (*P* values) associated with Supplementary Figure 1, Supplementary Figure 2, supplementary Figure 5 and Supplementary Figure 6 were calculated by performing a two-sample unequal variance student's *t*-test.

REFERENCES

1. Tabariès S, Dong Z, Annis MG, Omeroglu A, Pepin F, Ouellet V, Russo C, Hassanain M, Metrakos P, Diaz Z, Basik M, Bertos N, Park M, Guettier C, Adam R, Hallett M, et al. Claudin-2 is selectively enriched in and promotes the formation of breast cancer liver metastases through engagement of integrin complexes. Oncogene. 2011; 30:1318–1328.
2. Pfaffl MW. A new mathematical model for relative quantification in real-time RT-PCR. Nucleic Acids Res. 2001; 29:e45.
3. Deblois G, Chahrour G, Perry MC, Sylvain-Drolet G, Muller WJ, Giguere V. Transcriptional control of the ERBB2 amplicon by ERRalpha and PGC-1beta promotes mammary gland tumorigenesis. Cancer Res. 2010; 70:10277–10287.
4. Ikari A, Sato T, Watanabe R, Yamazaki Y, Sugatani J. Increase in claudin-2 expression by an EGFR/MEK/ERK/c-Fos pathway in lung adenocarcinoma A549 cells. Biochim Biophys Acta. 2012; 1823:1110–1118.
5. Rose AA, Annis MG, Dong Z, Pepin F, Hallett M, Park M, Siegel PM. ADAM10 releases a soluble form of the GPNMB/Osteoactivin extracellular domain with angiogenic properties. PLoS One. 2010; 5:e12093.

SUPPLEMENTARY FIGURES

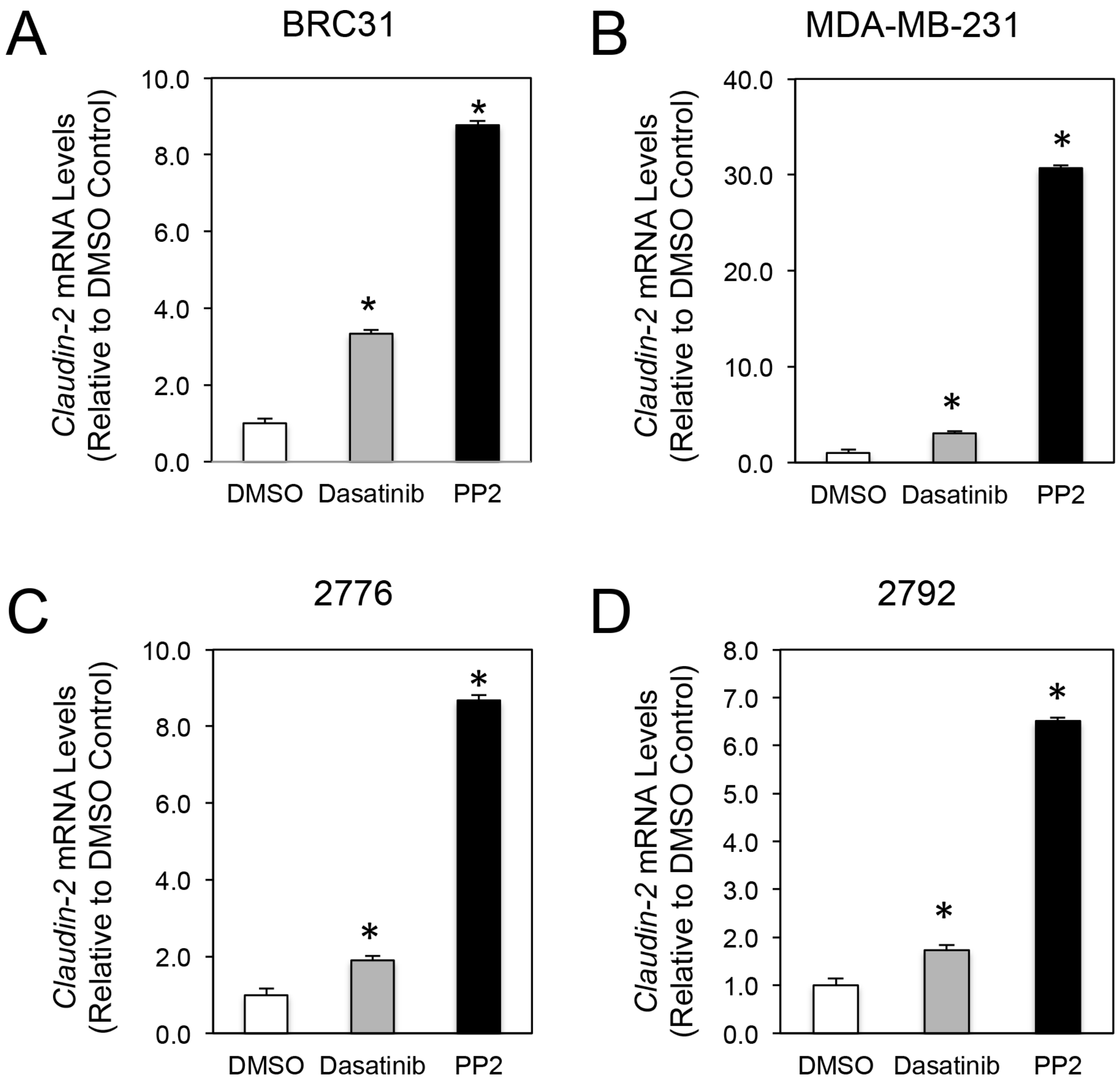

Supplementary Figure 1: Regulation of Claudin-2 expression by c-Src family kinase inhibitors occurs at the transcriptional level in breast cancer cells. Src-family kinase inhibitors induce expression of *Claudin-2* mRNA in human **(A** and **B)** and mouse **(C** and **D)** breast cancer cells (2776, 2792: liver-metastatic derivatives from 4T1 cell line). *, $P < 0.05$ (relative to vehicle control in all cases).

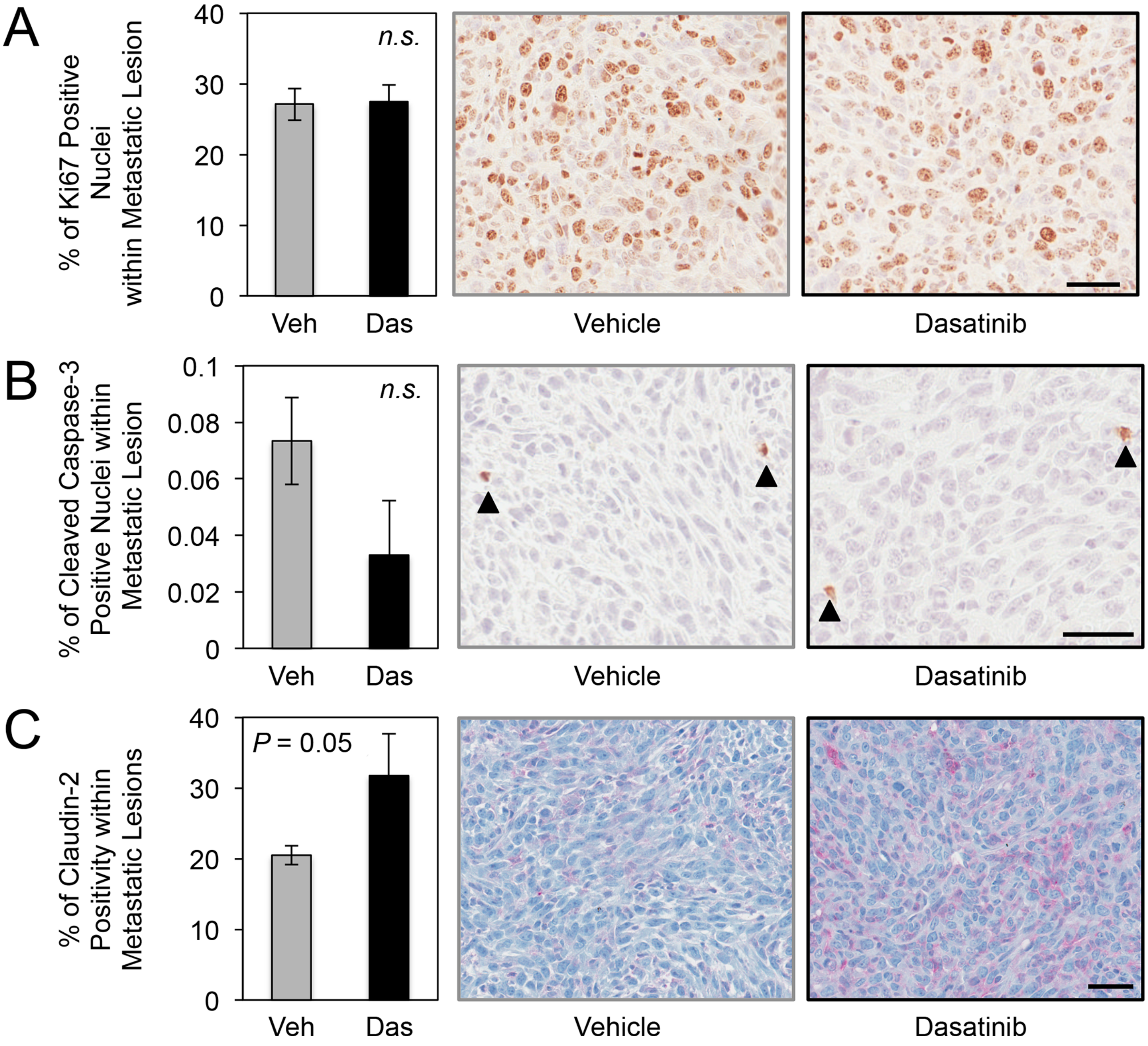

Supplementary Figure 2: Dasatinib treatment does not influence cell proliferation or apoptosis but induces Claudin-2 expression in breast cancer liver metastases. Livers, from the experiment described in Figure 3, were collected and subjected to immunohistochemical staining for a proliferation marker (Ki67) **(A)**, an apoptosis marker (Cleaved Caspase-3) **(B)** or Claudin-2 **(C)**. Data is quantified for each marker specifically within the liver metastases. No statistical differences for proliferation or apoptosis was observed within liver metastases that emerged in control (Par Veh) compared to Dasatinib-treated cohorts (Par Das). However, hepatic lesions in mice receiving Dasatinib possessed statistically significant increases in Claudin-2 expression compared to those in the control cohort. Scale bar (**a–c**) represents 40 µm. n.s., not significant.

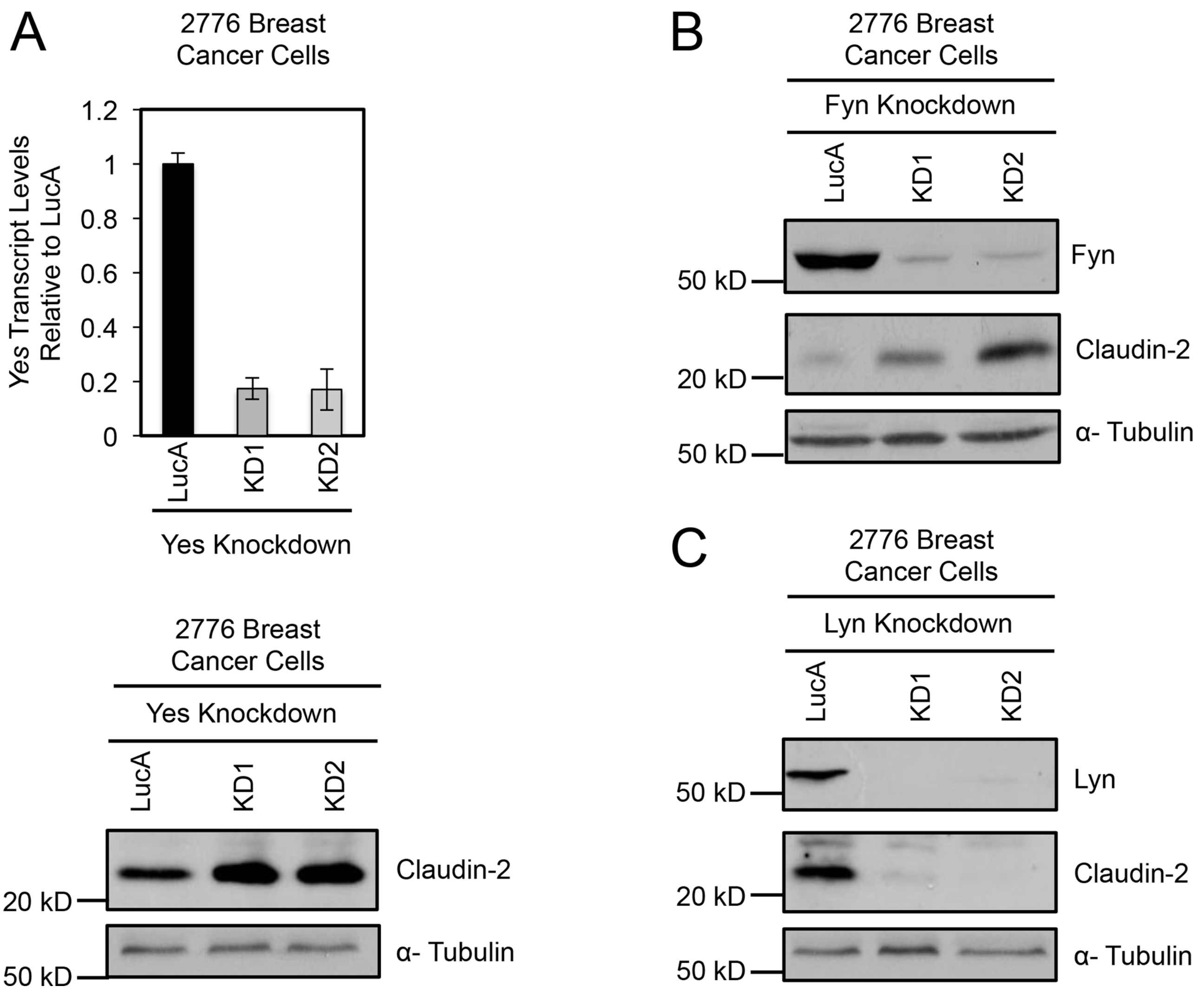

Supplementary Figure 3: Reduction of Yes or Fyn expression increases Claudin-2 levels, while reduced Lyn expression lowers Claudin-2 levels, in breast cancer cells. **(A)** Quantitative real-time PCR analysis was performed for *YES1,* normalized to total *Gapdh* levels, in 2776 liver-aggressive cell harbouring a control vector (LucA) or infected with two independent shRNA expression vectors (KD1 and KD2) against *Yes (upper panel)*. Immunoblot analysis for Claudin-2 in 2776 cells harboring individual Yes knockdowns versus LucA controls *(lower panel)*. Antibodies that recognize mouse Yes protein could not be identified. Immunoblot analysis of Claudin-2 expression in 2776 liver-aggressive cells harbouring a control vector (LucA) or infected with two independent shRNA expression vectors (KD1 and KD2) against Fyn **(B)** or Lyn **(C)**. As a loading control, total cell lysates were blotted for α-Tubulin.

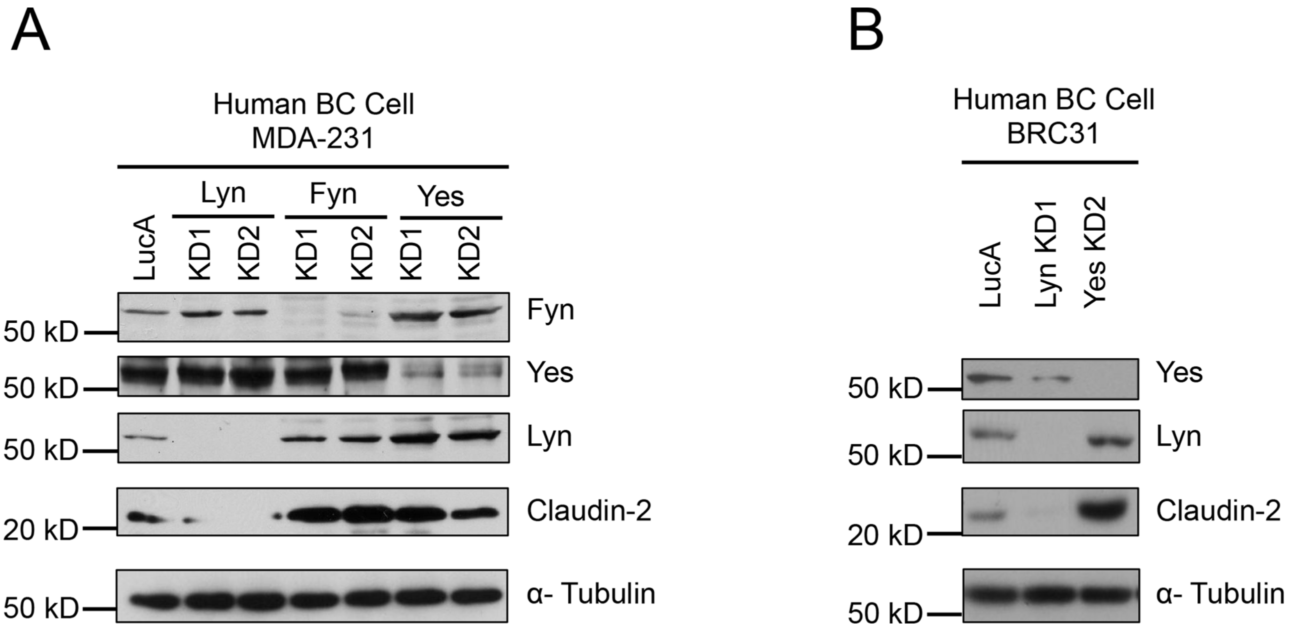

Supplementary Figure 4: Reduction of Fyn or Yes expression increases Lyn levels in breast cancer cells. Immunoblot analysis of Claudin-2 expression in both human MDA-MB-231 **(A)** and BRC31 **(B)** breast cancer cells harbouring a control vector (LucA) or infected with two independent shRNA expression vectors (KD1 and KD2) against Fyn, Yes or Lyn. As a loading control, total cell lysates were blotted for α-Tubulin.

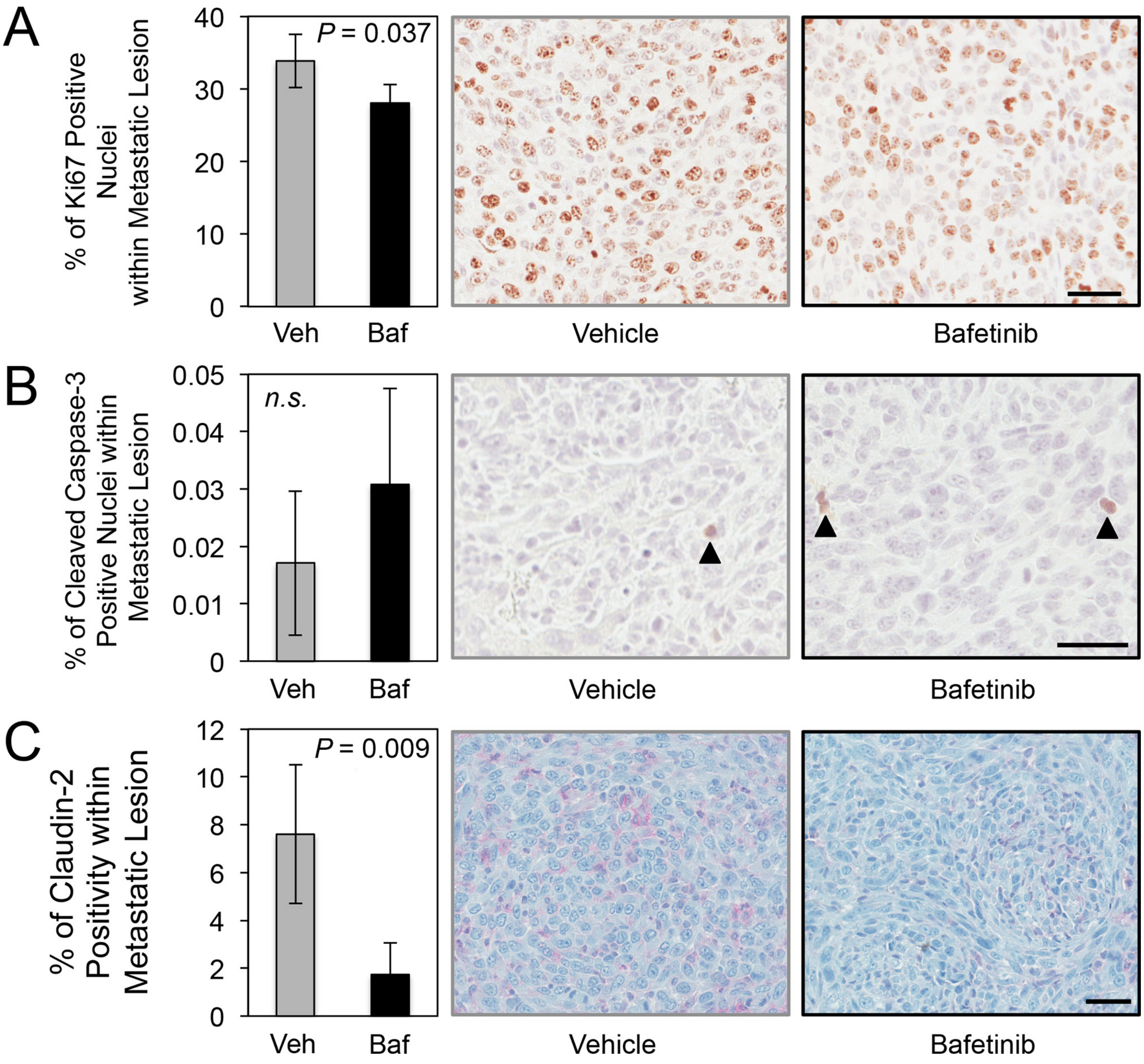

Supplementary Figure 5: Treatment with Bafetinib is associated with decreased proliferation and diminished Claudin-2 expression in liver metastases. Livers, from the experiment described in Figure 6, were collected and subjected to immunohistochemical staining for a proliferation maker (Ki67) **(A)**, an apoptotic marker (Cleaved Caspase-3) **(B)** or Claudin-2 **(C)**. Data is quantified for these individual markers specifically within the liver metastases. A statistically significant decrease in proliferation and in Claudin-2 positivity is observed in liver metastases from the Bafetinib-treated cohort (Par Baf) when compared to the control cohort. Scale bar indicated in each panel represents 40 μm. n.s., not significant.

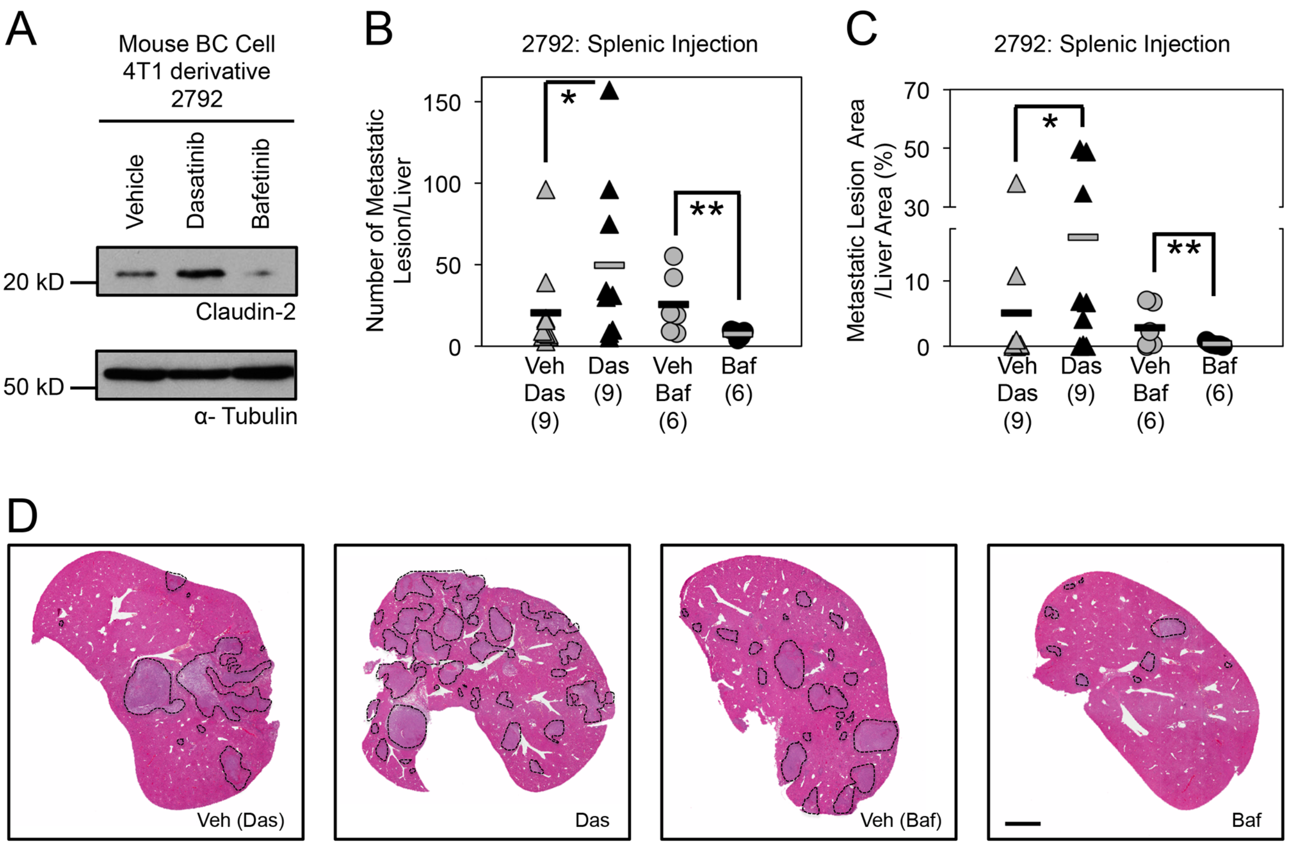

Supplementary Figure 6: Dasatinib treatment enhances, while Bafetinib treatment impairs, the formation of breast cancer liver metastases. **(A)** Immunoblot analysis of Claudin-2 expression in 2792 liver-aggressive breast cancer cells following treatment with either a pan-SFK inhibitor (Dasatinib) or a Lyn-selective inhibitor (Bafetinib). As a loading control, total cell lysates were blotted for α-Tubulin. **(B)** Dasatinib treatment increases both the number of liver metastases (*, $P < 0.03$) and overall liver-metastatic burden (*, $P < 0.032$) following splenic injection of 2792 liver-metastatic cells **(C)**. In contrast, the Bafetinib treated cohort exhibit fewer numbers of liver metastases (**, $P < 0.035$) **(B)** and a reduction in the overall liver metastatic burden (**, $P < 0.05$) compared to the vehicle control cohort following splenic injection of 2792 liver-metastatic cells (5×10^4 cells) **(C)**. The number of mice analyzed in each cohort is indicated in parentheses. **(D)** Representative images of H&E stained liver sections exhibiting the liver metastatic burden in each cohort. Scale bar represents 2mm and applies to all panels. Veh, Vehicle; Das, c-Src family kinase inhibitor (Dasatinib); Baf, Lyn-selective inhibitor (Bafetinib).
